# Does witnessing multitasking impact turnover and conflict? The influence of employee dark core

**DOI:** 10.1371/journal.pone.0290558

**Published:** 2023-09-20

**Authors:** Courtney L. Baker, Rushika De Bruin

**Affiliations:** 1 Department of Psychology, East Carolina University, Greenville, NC, United States of America; 2 Carilion Clinic, Roanoke, VA, United States of America; COMSATS University Islamabad - Wah Campus, PAKISTAN

## Abstract

This paper explores the dark core’s role in an employee’s evaluations of coworkers electronic multitasking behaviors. Using an experimental vignette design collected via Amazon’s Mechanical Turk (N = 485), we demonstrate that employees high in the dark core report higher turnover intentions and more interpersonal conflict, regardless of the multitasking behavior relevance. A three-way interaction between multitasking relevance, perceived intentionality, and the dark core when predicting turnover intentions emerged. Perceived coworker intentions played the largest role in impacting turnover and interpersonal conflict. Implications for theory and practice are discussed below.

## Introduction

The modern workplace has become a technology-saturated environment [1], with employees having to shift their interactions to a virtual format [2]. This allowing opportunities for coworkers to be evaluated for their technology use, especially when it differs from organizational norms or personal preferences [3]. Electronic multitasking, using technology to work on multiple tasks [4], is common practice within many workplaces and can be viewed negatively or positively [3]. There are personality and context-specific antecedents of engaging in electronic multitasking [5] However, research calls for the evaluation of electronic multitasking interacting with personality as it impacts coworker relationships and organizational outcomes [3, 6, 7]. For instance, polychronicity facilitates positive perceptions whereas trait anger inconsistently impacts perceptions of electronic multitasking [3]. These inconsistencies called for other more self-oriented personality traits to be evaluated. Therefore, the current paper focuses on other personality factors that impact electronic multitasking perceptions and organizational outcomes.

Dark personality has recently become a focus in research to explain everyday behaviors. The Dark Triad—narcissism, Machiavellianism, and psychopathy—are personality traits that facilitate negative workplace behaviors, such as workplace conflict [8] or counterproductive workplace behaviors (CWBs) [9]. Workers high in these personality traits can often be positively evaluated in larger workplace contexts (e.g., job performance) because they can be charming and assertive, which on the surface, make for good employees and managers [10, 11]. However, in the current age of teamwork and collaboration, the negative attributes of dark personality may come into play. Considering technology-saturated workplaces where employees are often using technology and engaging in electronic multitasking, the insensitivity and focus on themselves and their preferences of those who score higher on dark personality traits can be particularly influential in group-based situations and produce more workplace conflict [12, 13].

**Fig 1:**
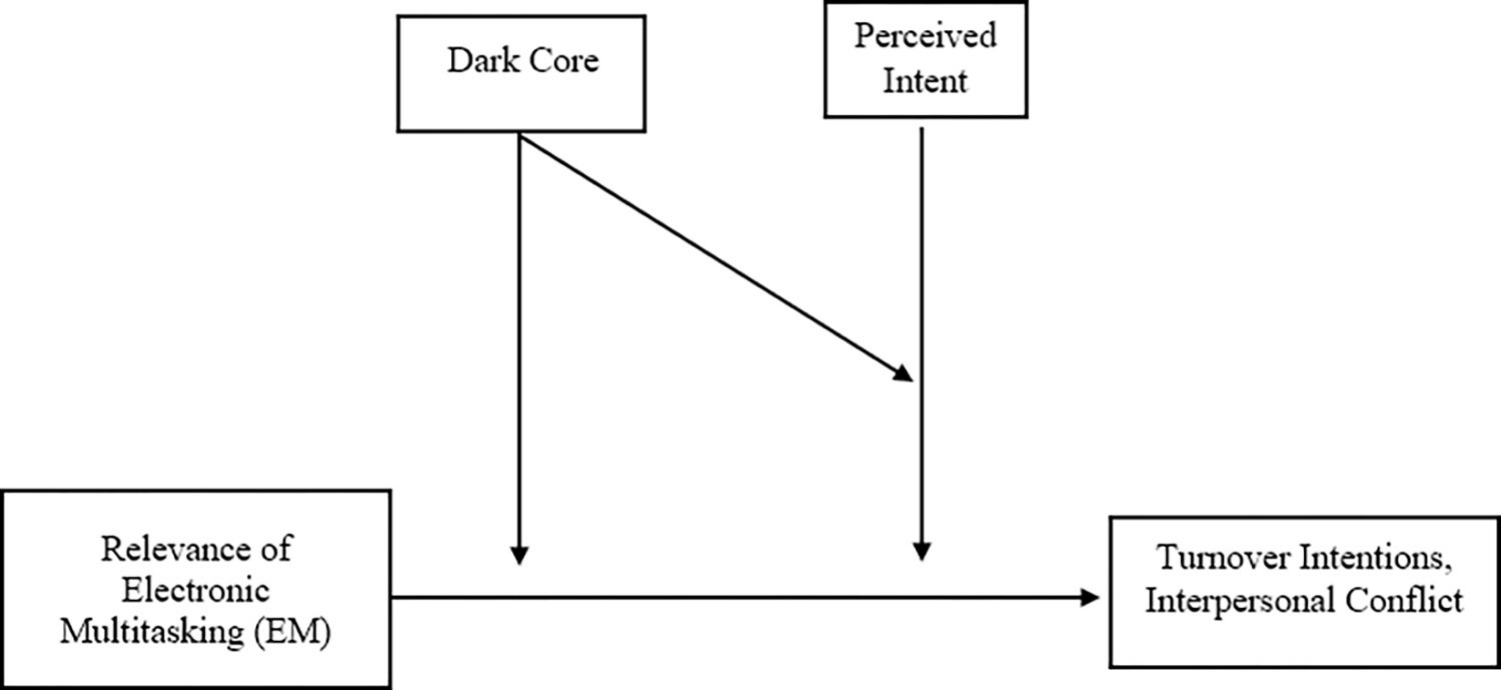
Theoretical model.

Contextual information (e.g., relevance of the secondary task, engagement in the primary task, task interdependence) also impacts reactions to multitasking [3, 7, 14]. Given that both personality variables and context individually impact employee reactions, the current paper explores dark personality within an electronic multitasking context and the role they both play in impacting employee turnover and interpersonal conflict. Thus, we evaluate boundary conditions of contextual features through the use of experimental vignettes to determine electronic multitasking reactions through the evaluation of the dark core, especially in everyday, neutral, and common situations (Fig 1).

The current paper makes three contributions to the current body of literature on workplace multitasking. First, the paper extends the outcomes beyond social evaluations [3] and answers calls of research to highlight the impact of coworker multitasking evaluations on organizational-oriented outcomes. Second, it highlights the role of dark personality as a contributing factor to the relationship between multitasking contextual features and organizational outcomes. We theoretically articulate why those with a “dark core” may be particularly vulnerable when evaluating coworker multitasking behaviors. Third, we highlight the elusive role that contextual features play in understanding multitasking perceptions. While prior research highlights secondary task relevance and synchronicity, we highlight boundary conditions in which this might not emerge. Finally, this paper fills the gap in the literature by integrating the research on multitasking social evaluations and contextual features of multitasking [3] with work on workplace outcomes and the role of observer personality, which to the authors knowledge has not been done before.

### Dark core in the workplace

By focusing on contextual features, researchers ignore a large aspect that shapes employee perceptions of electronic multitasking, their personality [15]. Two employees may find themselves in similar workplace situations, like witnessing their colleague multitask, but react differently because of their personality profile. One theoretical perspective that highlights the situation and personality interaction as highly important is the Trait Activation Theory (TAT) [16]. TAT posits that some contexts activate employee personality traits that may not always be active and justify certain employee reactions. Thus, threatening situations may activate more harmful traits that are not generally prevalent in the workplace. However, each person’s perception of “threatening” varies, based on the situational cues that the employee perceives and their personality profiles. We specifically chose the darker traits instead of the commonly explored Big Five (e.g., brighter traits) because negative situations tend to activate negative behavioral outcomes when they interact with darker traits [17]. Electronic multitasking can be conceptualized as a negative workplace situation with some employees evaluating it as uncivil [7] and rude [3].

Though the dark personality traits are categorized as theoretically distinct, there is overlap between them, characterized as the dark core, driven by the tendency towards socially, morally, or ethically questionable behaviors [18]. Individuals who score high on the dark core engage in behaviors that maximize their own utility, regardless of the cost to others, while continuously justifying their behaviors. The shared lack of empathy, or callousness, towards others and high levels of self-interest facilitates this process [19]. Reports indicate that using the dark core is a more parsimonious account of employee personality unless there is a particular reason to expect their unique trait to become relevant [20]. Employees with these traits impact workplace interactions [21], especially in a world where collaborations and teamwork are encouraged [22].

The dark personality traits that make up the dark core are subclinical levels of narcissism, Machiavellianism, and psychopathy [19]. Narcissists have higher expectations about career success [23] and perceive that they offer more workplace value [24]. Machiavellians are manipulative and have no qualms about deceiving or exploiting others to achieve their own goals [25]. They focus on short term profit maximization, even at the detriment of interpersonal relationships [26] and react more negatively in uncertain and stressful situations [27], which is one way to characterize a norm-violating situation, like electronic multitasking. Psychopathy has been linked to negative reactions to workplace stressors [28]. While these traits may be characterized as toxic, employees with various levels are abundant [21] as they often have good impression management skills [10].

### Organizational outcomes of electronic multitasking

As more people use electronic devices, employees in group situations are forced to renegotiate interpersonal behavior norms [29]. Multitasking in a meeting is disrespectful towards the individual who is speaking [30] indicating that electronic multitasking can be judged negatively if undertaken for what coworkers believe is the wrong reason. Witnessing coworkers engage in irrelevant behavior can lead to non-favorable evaluations like perceiving that coworker as ruder, less agentic, or less communal [3]. This is especially true if employees are already predisposed to perceive negativity based on their personality.

With the growing use of electronic multitasking, there have been calls for research to understand what the social implications are and how coworker relationships are impacted [14, 31]. This led to the exploration of social consequences associated with coworker electronic multitasking [3, 7, 14]. However, to our knowledge, there has not been an empirical evaluation of electronic multitasking on organizational outcomes. While the Dark Triad impacts some organizational behaviors (e.g., interpersonal conflict, CWBs), it has not been explored in mundane situations that employees face. The aspects that impact perceptions (i.e., context and personality) can also impact organizational outcomes. Thus, this paper focuses on one common context: using laptops in a work meeting and posits that witnessing these behaviors impact workplace outcomes.

#### Turnover intentions

Using Conservation of Resources Theory as a lens with which to explain the relationship between electronic multitasking and turnover, having positive relationships and coworker support act as resources that buffer against job-related stress, reducing turnover intentions [32]. Coworker support plays a protective role in alleviating emotional exhaustion and impacts turnover intentions [33] underscoring the importance that coworkers play in turnover intentions. Electronic multitasking, a common CWB, influences whether an employee will like their coworkers and further facilitate that employee’s workplace experiences. Negative coworker relationships that stem from witnessing irrelevant multitasking behaviors are more likely to increase the likelihood that employees look for other jobs.

#### Interpersonal conflict

To better understand the role of conflict in multitasking situations, Social Exchange Theory (SET) [34] notes that employees form high quality relationships following positive transactions. Conversely, coworkers engaging in negative behaviors in a social exchange can be detrimental to those relationships and lead to subsequent interpersonal conflict, characterized by negative social coworker interactions caused by evaluating their behaviors undesirably [35]. Compared to other workplace stressors, employees were most likely to complain about interpersonal conflict [36]. Evaluating electronic multitasking as uncivil leads to more voicing behaviors, meaning that employees are more likely to indicate that they are unhappy with the behavior itself [37], potentially leading to more conflict. Even though organizationally encouraged in some situations because of the benefits, multitasking in a group situation can be evaluated as violating group norms, especially when irrelevant to the topic at hand [14], leading to misunderstandings and more interpersonal conflict [31].

*Hypothesis 1*. Relevant multitasking will predict (a) lower turnover intentions and (b) less interpersonal conflict compared to irrelevant multitasking.

While contextual information (e.g., multitasking relevance) may impact downstream behaviors, an interactionist perspective dictates that situational factors alone are not sufficient to predict workplace behaviors. Rather, drawing from TAT, specific contexts activate personality traits, such as the dark core. Being subject to negative interactions from a coworker lead to CWBs when employees have high levels of Dark Triad [38]. Employees who were mistreated by their peers were more likely to engage in CWBs if they were higher in narcissism, as those situations triggered employees to react negatively when they feel like they had been wronged [39]. Additionally, most individuals who are higher in dark personality tend to avoid commitment [21]. When exposed to norm violating situations, they may not have the same attachments that encourage them to stay. As electronic multitasking violates norms, those with higher dark core rates may engage in more conflict and turnover as a reaction.

*Hypothesis 2*. The dark core strengthens the negative relationship between irrelevant multitasking and (a) turnover and (b) interpersonal conflict.

#### Intent

Another factor that could impact employee behaviors is perceived coworker intentionality. Drawing from incivility research, coworkers can violate norms, like engaging in irrelevant multitasking, either with or without intent [40], which impacts employee behaviors. Intent is based on the individual’s choice to engage in such behaviors and is related to the subsequent consequences [41]. This intentionality may interact with the behaviors observed to predict an employee’s desire to leave that situation through exiting the organization or engage in conflict with the norm violating individual. Given the propensity of employees who are higher in the dark core to prioritize their own self-interests, situations associated with harm or violations can elicit more negative behavioral responses [42].

*Hypothesis 3*. Intent will strengthen the negative relationship between irrelevant multitasking and (a) turnover and (b) interpersonal conflict.
*Research Question*. Is there an interaction between the dark core and intent in moderating the relationship between multitasking and (a) turnover and (b) interpersonal conflict?

## Methods

### Participants

Participants were recruited from Amazon’s Mechanical Turk in June of 2020; the only identifying feature of the workers collected was their Mturk IDs and demographic variables. All procedures were approved by the Institutional Review Board. Eligibility criteria for the current study included being at least 18 years old and employed fulltime. An a priori G*Power indicated a sample size of 486 would be sufficient for this study (linear multiple regression with *f*^*2*^ = .03, α = .05, 1-β = .80, with 7 tested predictors, and 8 total number of predictors collected).

Based on the above criteria, 586 participants were recruited from Amazon’s Mechanical turk. A final sample of 485 participants were retained after accounting for data quality issues using Mahalanobis distance and longstring analysis (*n* = 101) [43]. More than half the sample identified as male (54.43%), a majority (73.61%) identified as White/Caucasian, ages ranged from 18–82 (*M* = 37.38, *SD* = 11.94), and were employed, at least part-time (Full time = 78.14%, part-time = 14.02%).

### Procedures

All procedures outlined in this study were approved by the Institutional Review Board at Northern Illinois University. After reading the recruitment materials, participants clicked on a Qualtrics link that took them to an informed consent document. In place of a written signature of consent, participants were asked to indicate that they agreed to participate or not based on the informed consent they read. After providing consent, participants were randomly assigned to read one of four vignettes [3] and evaluated the behaviors. They differed based on relevance (relevant: sending emails to other members of the creative team about product information versus irrelevant: sending emails to friends to plan an upcoming happy hour) and level of engagement (concurrent: *while* participating in the discussion versus sequential: *instead of* participating in the discussion). Participants rated perceived intent [44] and the extent to which they would engage in conflict [45] and turnover [46] because of the situation and provided ratings of their own dark core [47] and demographics.

### Measures and manipulations

Vignettes were used to manipulate electronic multitasking and were adapted from prior literature [3]. Participants read either a concurrent-irrelevant multitasking condition (colleagues were surfing the web and emailing friends about an upcoming happy hour while participating in the discussion), concurrent-relevant (colleagues were surfing the web and sending emails to their teammates while participating in the discussion), sequential-irrelevant (colleagues were surfing the web and emailing friends about an upcoming happy hour instead of participating in the discussion), and sequential-relevant (colleagues were surfing the web and sending emails to their teammates instead of participating in the discussion).

The dark core was assessed with the Dirty Dozen [47] with 12 items on a scale of 1 (*strongly disagree*) to 5 (*strongly agree*). The scale was found to be reliable (*α* = 0.95) and valid (see correlations in Table 1).

**Table 1:** Means, standard deviations, and correlations with confidence intervals.

Variable	Descriptive Statistics		Correlations		
	*M*	*SD*	1	2	3
1. Intent	1.93	0.88			
2. Core	3.19	1.08	.61**		
			[.55, .67]		
3. Conflict	3.10	1.16	.60**	.77**	
			[.53, .65]	[.73, .81]	
4. Turnover	3.11	1.21	.60**	.75**	.77**
			[.54, .66]	[.70, .78]	[.73, .80]

*Note*. *M* and *SD* are used to represent mean and standard deviation, respectively. Values in square brackets indicate the 95% confidence interval for each correlation. The confidence interval is a plausible range of population correlations that could have caused the sample correlation.

* indicates *p* < .05.

** indicates *p* < .01.

Interpersonal conflict was evaluated with 4 items [45] in which participants were asked to indicate their behavioral frequency on a scale of 1 (*never*) to 5 (*very often*). It includes questions such as “How often do you get into arguments with others at work?”. The scale was found to be reliable (*α* = 0.89) and valid (see correlations in Table 1).

Turnover intentions were assessed with 3 items [46] asking participants to indicate the frequency that they engaged in each behavior on a scale of 1 (*never*) to 5 (*always*) if they were at the job where they observed the coworker described. It includes items such as “It is very likely that I will leave my job”. The scale was found to be reliable (*α* = 0.85) and valid (see correlations in Table 1).

This was measured with a single item which asked participants “Was harm intended by the coworker” [44]. Responses included 1 (no intent to harm), 2 (ambiguous intent to harm), and 3 (clear intent to harm).

Finally, age, gender, ethnicity, and employment status was assessed for all participants.

### Analysis strategy

Independent samples t-tests were used to explore if there are differences between the multitasking relevance sample and the multitasking irrelevant sample on turnover intentions and interpersonal conflict. We further used multiple linear regression to estimate the relationship between personality and multitasking relevance or intent and each organizational outcome. This was used to measure how strong the relationship is between each independent variable and the turnover outcomes and interpersonal conflict. All data can be found here: https://osf.io/mz5bx/?view_only=551ece9d76094ca0b6033065ae263487

## Results

### Hypothesis testing

All measured variables scale scores were calculated through an aggregate of the items. Means and correlations for all variables are shown in Table 1. All available data were used. Two independent samples t-tests were run to evaluate hypothesis 1. There were no significant differences between multitasking relevance in predicting turnover intentions, *t*(482.99) = 0.45, *p* = .656 or interpersonal conflict, *t*(479.44) = 0.59, *p* = .557, failing to support hypothesis 1.

We explored if either the dark core or intent predicted each outcome using a multiple linear regression. The first model tested turnover intentions (*F* (3,466) = 225.00, *p* < .001, *R*^*2*^ = 0.59; Table 2). The dark core predicted more turnover intentions (β = 0.49, *p* < .001). However, neither intent (β = 0.05, *p* = .698) nor the interaction between dark core and intent were significant (β = 0.27, *p* = .131). The second model tested interpersonal conflict (*F* (3,465) = 267.10, *p* < .001, *R*^*2*^ = 0.63; Table 2). Similarly, the dark core predicted more interpersonal conflict (β = 0.62, *p* < .001), and neither intent (β = 0.11, *p* = .359) nor the interaction between intent and the dark core (β = 0.12, *p* = .493) predicted interpersonal conflict.

**Table 2:** Regression results for turnover and conflict predicted by the dark core and intent.

	Turnover				Conflict			
Predictor	β	*SE*	*t*	*p*	β	*SE*	*t*	*p*
Core	0.49	0.09	5.98	< .001	0.62	0.08	7.90	< .001
Intent	0.05	0.17	0.39	.698	0.11	0.16	0.92	.359
Core x Intent	0.27	0.05	1.51	.131	0.12	0.04	0.69	.493
Fit	*F* (3,466) = 225.00, *R*^*2*^ = .589, *p* < .001				*F* (3,465) = 267.10, *R*^*2*^ = .630, *p* < .001			

*Note*. This table represents a multiple linear regression without the presence of the condition participants were assigned.

To evaluate hypothesis 2, we ran two multiple linear regressions where relevance and the dark core were added as independent variables and turnover and interpersonal conflict were the outcome variables. When evaluating turnover intentions (*F* (3,481) = 202.40, *p* < .001, *R*^*2*^ = 0.56), relevance was not impactful (β = 0.01, *p* = .847), but the dark core predicted more turnover intentions (β = 0.77, *p* < .001). The interaction was non-significant (β = -0.03, *p* = .808). The second model tested interpersonal conflict (*F* (3,480) = 237.60, *p* < .001, *R*^*2*^ = 0.60). While relevance did not predict interpersonal conflict (β = -0.12, *p* = .203), the dark core predicted more interpersonal conflict (β = 0.67, *p* < .001). However, the interaction was non-significant (β = 0.15, *p* = .223). As both interactions were not significant, we did not have support for hypothesis 2, but we do see the impact the dark core has on behaviors.

Finally, to evaluate hypothesis 3, we ran two multiple linear regressions where relevance and intent were added as independent variables and turnover and interpersonal conflict were the outcome variables respectively. The first model tested the impact of relevance and intent on turnover intentions (*F* (3,466) = 88.00, *p* < .001, *R*^*2*^ = 0.36). Like the prior analyses, while multitasking condition did not predict turnover intentions (β = -0.04, *p* = .675), intent predicted more turnover intentions (β = 0.55, *p* < .001). However, the interaction was not significant (β = 0.07, *p* = .621). The second model tested the impact on interpersonal conflict (*F* (3,465) = 86.68, *p* < .001, *R*^*2*^ = 0.36). While multitasking condition did not predict interpersonal conflict (β = -0.15, *p* = .098), intent predicted more interpersonal conflict (β = 0.42, *p* < .001). However, the interaction was not significant (β = 0.23, *p* = .109). Thus, we did not have support for hypothesis 3, but we see the impact of attributing intentionality on behavioral outcomes.

### Exploratory research question

**Fig 2:**
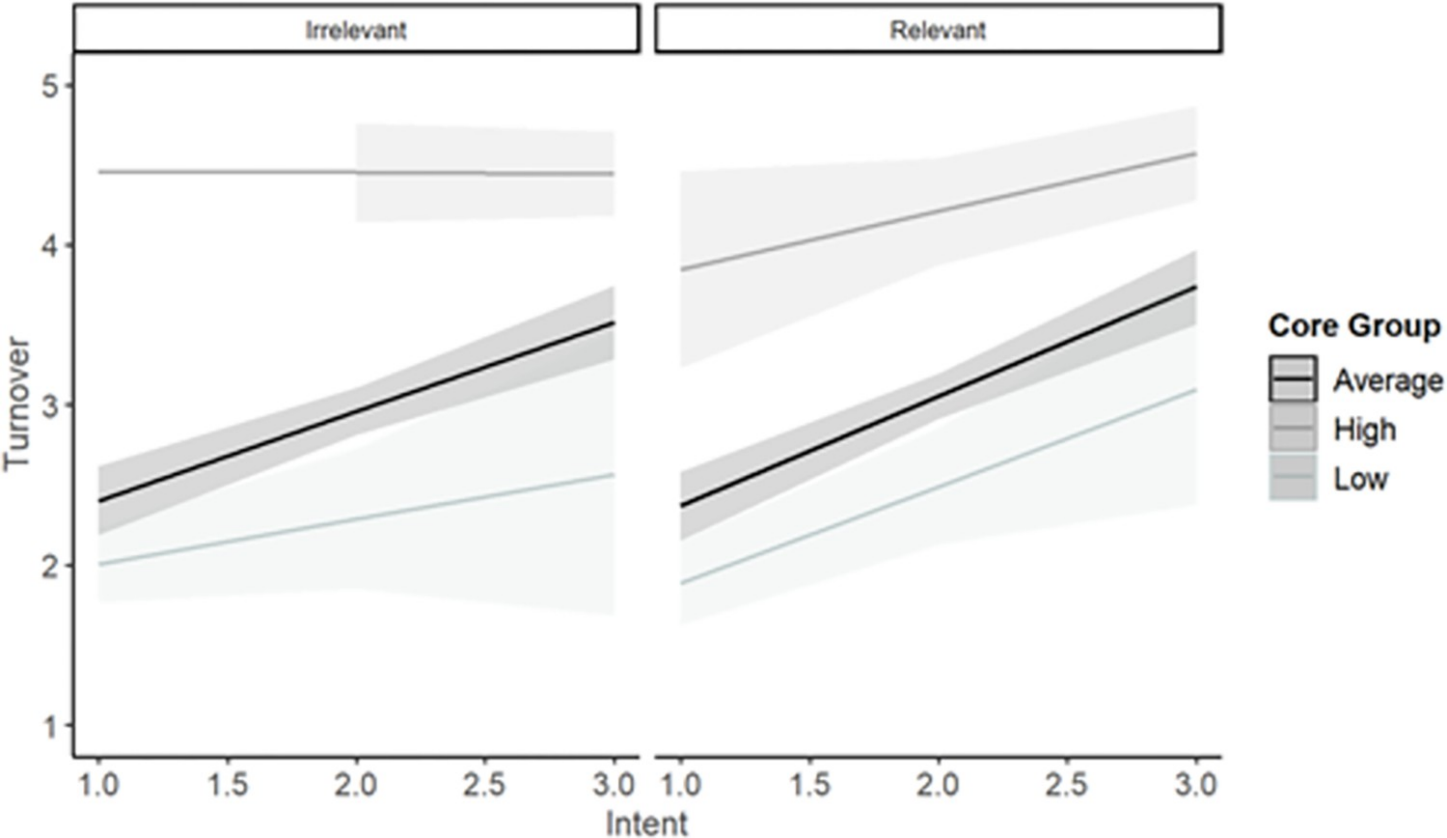
Three-way interaction of intent, the dark core, and multitasking relevance predicting turnover.

The final set of analyses explored the three-way interaction between intent and the dark core in moderating the relationship between multitasking condition and turnover and interpersonal conflict. In the model evaluating turnover intentions, (*F* (7,462) = 97.60, *p* < .001, *R*^*2*^ = 0.60; see Table 3), the three-way interaction was significant. Simple slopes revealed at low levels of the dark core, there was a difference between relevance conditions in the impact of intent on turnover intentions where for the relevant condition, the relationship between intent and turnover was non-significant (β = 0.01, *p* = .911), but there was a positive relationship for the irrelevant multitasking condition (β = 0.41, *p* < .001). This indicates that when employees are lower in the dark core, intentionality may play a larger impact in the relationship between secondary task relevance and turnover intentions. At high levels of the dark core, regardless of intent, electronic multitasking leads to higher turnover, whereas this is not the same with those low or average in the dark core where intentionality plays a larger role. However, at high levels of the dark core, the relationship between intent and turnover intentions was similar for both relevant multitasking (β = 0.38, *p* < .001) and irrelevant multitasking (β = 0.37, *p* < .001; Fig 2). That is, regardless of electronic multitasking relevance, those who perceive high intentionality from their coworkers were more likely to leave.

**Table 3:** Regression results for turnover and conflict with 3-way interaction.

	Turnover				Conflict			
Predictor	β	*SE*	*t*	*p*	β	*SE*	*t*	*p*
Relevance (Irrelevant)	0.44	0.57	1.85	.065	0.35	0.52	1.57	.117
Core	0.60	0.13	5.12	< .001	0.68	0.12	6.04	< .001
Intent	0.32	0.24	1.84	.067	0.29	0.22	1.72	.086
Relevance (Irrelevant) x Core	-0.38	0.19	-1.39	.164	-0.19	0.17	-0.75	.456
Relevance (Irrelevant) x Intent	-0.76	0.34	-2.28	.023	-0.48	0.31	-1.53	.128
Core x Intent	-0.07	0.07	-0.27	.791	-0.03	0.06	-0.13	.896
Relevance (Irrelevant) x Core x Intent	0.74	0.10	1.98	.049	0.33	0.09	0.93	.352
Fit	*F* (7,462) = 97.60, *R*^*2*^ = .591, *p* < .001				*F* (7,461) = 116.50, *R*^*2*^ = .633, *p* < .001			

*Note*. This table represents a multiple linear regression with the presence of the condition participants were assigned and three-way interaction term.

The model evaluating interpersonal conflict (*F* (7,461) = 116.50, *p* < .001, *R*^*2*^ = 0.64; Table 3). The three-way interaction was not significant (β = -0.32, *p* = .352). Of all the variables included, only the dark core predicted interpersonal conflict (β = 0.56, *p* < .001).

## Discussion

With the growing use of workplace technology and increasing team-oriented projects, employees are witnessing coworkers multitasking using information communication technologies (ICTs) now more than ever before. Multitasking behaviors are evaluated depending on their characteristics, such as relevance [3, 14], and we integrate another important aspect, employee dark personality, that could strongly impact employee behaviors. The study explores behavioral and distal effects of coworker electronic multitasking behaviors that have not been explored.

### Multitasking and personality impact on organizational outcomes: Empirical contributions

Multitasking impacts employee social interactions [7] and positive coworker relationships encourage commitment behaviors and favorable reactions. Others also found that multitasking acts as a stressor in today’s technology saturated environment where it is more common because of the virtual environment [2]. Therefore, it was surprising that the current study showed no differences in either turnover intentions or interpersonal conflict based on the relevance of the secondary task coworkers were engaging in and did not support hypothesis 1. However, reading about a single interaction may not have the same impact on behavioral responses like social evaluations because technology use has become so common in the current workplace [1]. Seeing a coworker repeatedly violating workplace and group norms may lead to stronger behavioral responses. Alternatively, the impact of electronic multitasking on organizational behaviors could need to go through social implications, rather than having a direct effect. Future research should explore this as a mechanism.

The dark core had a strong effect on organizational outcomes where those higher in the core reported higher turnover intentions and more interpersonal conflict. Given that this study used a common workplace behavior that employees could come across in the present technology-saturated workplace, it was important to consider how employees react to such behaviors. The high level of self-interest shared between the traits implies that coworker electronic multitasking could be perceived as threatening and could lead employees to more conflict with those who have wronged them and eventually exit the situation. Interestingly, the dark core predicted organizational outcomes over and above the provided contextual information, which was not anticipated in Hypothesis 2. Regardless of multitasking relevance, the dark core predicted more turnover and conflict. This could be because personality is more influential than singular mundane situations. Future research should explore these relationships in a more organic setting with established relationships to explore repeated norm violations along with different degrees of violation. For instance, maybe multitasking is not an egregious violation, whereas speaking loudly and disrupting the discussion would be a more serious violation that could have stronger impacts.

We confirm what has been found in the past where employees higher in dark personality tend to engage in more negative workplace behaviors like increased interpersonal conflict [8] or turnover as suggested by hypothesis 3 and our exploratory research question. Pulling from Trait Activation Theory [15], we expected situational information, multitasking relevance, to activate the dark core and justify engaging in more negative workplace behaviors. However, we did not find evidence for such an effect with the specific context we used in this study. The context was possibly not sufficiently threatening to justify a stronger reaction from employees higher in the dark core because of the ordinariness. Future studies should evaluate other everyday potentially threatening contexts, or repeated behaviors that employees tend to be exposed to frequently.

### Theoretical contributions

Theoretically, the current study adds to the existing literature on the interaction between personality and situation in driving employee responses. As Trait Activation Theory suggests [15], the interaction between the dark core personality, the situational context features of relevance, and the intent to harm drive the responses for turnover and conflict at work. This suggests that work considering personality variables in workplace settings should consider the role the situation plays in facilitating these relationships as well and whether the two interact to impact each other.

### Limitations and future directions

This paper takes the next step in answering questions regarding workplace outcomes associated with electronic multitasking behaviors, the new norm, and the interaction with darker personality, there are a few limitations that must be addressed. First, all the scales were assessed in one survey asked from a single respondent which can be associated with common method bias [48]. However, because this study focuses on an employee’s perceptions about their coworker, this self-report method is appropriate and does not inflate relationships simply because of the collection method [49]. This is also corroborated within organizational research [50] reporting that the impact of common method bias tends to be oversimplified and researchers should instead focus on any measurement biases. We also mitigated these effects by providing detailed explanations of the purpose and nature of the measures to emphasize anonymity of responses. Respondents were also given the option to leave at any point in the survey if they felt uncomfortable [51]. While we isolated the impact of electronic multitasking through vignettes which are an appropriate method to measure common, specific workplace outcomes [52, 53], researchers should explore if these effects replicate in organizations. These vignettes have been used in past research to measure similar types of social judgements [3, 14, 37]. This also allows researchers to explore the impact of organizational norms. We echo the call for future research to evaluate electronic multitasking’s influence on workplace norms [3]. While electronic multitasking is perceived as a norm violation [14], researchers could evaluate norm renegotiations in this technology-driven world and how electronic multitasking plays a role in that.

Future research could expand the current work to employee productivity. Multitasking and productivity is generally negatively related and some introductory work has looked at the impact of the Dark Triad on productivity. Research should explore the interaction of these and the impact on productivity because of the importance that organizations place on employee productivity. Coworker electronic multitasking can act as a distraction and reduce employee productivity, but this should be empirically tested. This would be especially impactful if those high in the dark core feel slighted by the norm violation and thus withdraw their own efforts from the organization and team.

While this study explores some common contextual factors that impact employee perceptions and behaviors, it did not focus on employee or coworker demographic factors. Future research should evaluate the impact on perceptions as past research indicates that minority members perceive more negativity in threatening situations [54]. Thus, the interaction of demographic factors along with personality may point to differing organizational outcomes.

## Conclusion

The current study adds to the body of literature exploring the social evaluations of electronic-based multitasking and answers calls of research to evaluate the impact these evaluations have on workplace outcomes, like conflict and turnover. While these social evaluations do not directly inform the outcomes under investigation, it could be that other important workplace outcomes (e.g., trust and liking with coworkers) might be impacted. However, the current study illuminates the role that the dark core plays in changing the relationships between situational features and employee outcomes when considering social evaluations of coworker intent. This combines the area of personality and technology-based multitasking. This can assist in the development of future work interventions regarding the use of multitasking in the workplace, and particularly within meetings.

## Supporting information

S1 Checklist PLOS ONE clinical studies checklist. (DOCX)

S2 Checklist STROBE statement—checklist of items that should be included in reports of observational studies. (DOCX)
