# Supplementary material for: Does witnessing multitasking impact turnover and conflict? The influence of employee dark core
Source: PLoS One. 2023 Sep 20;18(9):e0290558. doi: 10.1371/journal.pone.0290558 (PMC10511128; doi:10.1371/journal.pone.0290558)
Supplement: S2 Checklist — (DOCX) [file pone.0290558.s002.docx]

STROBE Statement—checklist of items that should be included in reports of observational studies

|  | Item No. | Recommendation | Page  No. | Relevant text from manuscript |
| --- | --- | --- | --- | --- |
| **Title and abstract** | 1 | (*a*) Indicate the study’s design with a commonly used term in the title or the abstract | 2, line 16 | Using an experimental vignette design collected via Amazon’s Mechanical Turk (N= 485), |
|  |  | (*b*) Provide in the abstract an informative and balanced summary of what was done and what was found | 2, line 15 - 22 | This paper explores the dark core’s role in an employee’s evaluations of coworkers electronic multitasking behaviors. Using an experimental vignette design collected via Amazon’s Mechanical Turk (N= 485), we demonstrate that employees high in the dark core report higher turnover intentions and more interpersonal conflict, regardless of the multitasking behavior relevance. A three-way interaction between multitasking relevance, perceived intentionality, and the dark core when predicting turnover intentions emerged. Perceived coworker intentions played the largest role in impacting turnover and interpersonal conflict. Implications for theory and practice are discussed below. |
| Introduction | | | |  |
| Background/rationale | 2 | Explain the scientific background and rationale for the investigation being reported | Pg 3 – 9, lines 25 – 178 |  |
| Objectives | 3 | State specific objectives, including any prespecified hypotheses | Figure 1 (theoretical model)  Lines 148 – 149 (Hypothesis 1); 162 – 163 (Hypothesis 2); 175 – 178 (Hypothesis 3 and Research Question 1) | *Hypothesis 1.* Relevant multitasking will predict (a) lower turnover intentions and (b) less interpersonal conflict compared to irrelevant multitasking.  *Hypothesis 2.* The dark core strengthens the negative relationship between irrelevant multitasking and (a) turnover and (b) interpersonal conflict.  *Hypothesis 3*. Intent will strengthen the negative relationship between irrelevant multitasking and (a) turnover and (b) interpersonal conflict.  *Research Question.* Is there an interaction between the dark core and intent in moderating the relationship between multitasking and (a) turnover and (b) interpersonal conflict? |
| Methods | | | |  |
| Study design | 4 | Present key elements of study design early in the paper | 56-58  Methods (Lines 179 – 219) | Thus, we evaluate boundary conditions of contextual features through the use of experimental vignettes to determine electronic multitasking reactions through the evaluation of the dark core, especially in everyday, neutral, and common situations (Fig 1). |
| Setting | 5 | Describe the setting, locations, and relevant dates, including periods of recruitment, exposure, follow-up, and data collection | Methods (Lines 179 – 219) | Participants were recruited from Amazon’s Mechanical Turk in June of 2020; the only identifying feature of the workers collected was their Mturk IDs and demographic variables. All procedures were approved by the Institutional Review Board. Eligibility criteria for the current study included being at least 18 years old and employed fulltime. An a priori G*Power indicated a sample size of 486 would be sufficient for this study (linear multiple regression with *f^2^*= .03, α= .05, 1-β= .80, with 7 tested predictors, and 8 total number of predictors collected). |
| Participants | 6 | (*a*) *Cohort study*—Give the eligibility criteria, and the sources and methods of selection of participants. Describe methods of follow-up  *Case-control study*—Give the eligibility criteria, and the sources and methods of case ascertainment and control selection. Give the rationale for the choice of cases and controls  *Cross-sectional study*—Give the eligibility criteria, and the sources and methods of selection of participants | Participants and Procedures (Lines 181 – 200) | **Participants**  Participants were recruited from Amazon’s Mechanical Turk in June of 2020; the only identifying feature of the workers collected was their Mturk IDs and demographic variables. All procedures were approved by the Institutional Review Board. Eligibility criteria for the current study included being at least 18 years old and employed fulltime. An a priori G*Power indicated a sample size of 486 would be sufficient for this study (linear multiple regression with *f^2^*= .03, α= .05, 1-β= .80, with 7 tested predictors, and 8 total number of predictors collected).  Based on the above criteria, 586 participants were recruited from Amazon’s Mechanical turk. A final sample of 485 participants were retained after accounting for data quality issues using Mahalanobis distance and longstring analysis (*n*= 101; Meade & Craig, 2012). More than half the sample identified as male (54.43%), a majority (73.61%) identified as White/Caucasian, ages ranged from 18 – 82 (*M* = 37.38, *SD* = 11.94), and were employed, at least part-time (Full time = 78.14%, part-time = 14.02%).  **Procedures**  Participants read one of four vignettes (adapted from De Bruin & Barber, 2019) and evaluated the behaviors. They differed based on relevance (relevant: sending emails to other members of the creative team about product information vs irrelevant: sending emails to friends to plan an upcoming happy hour) and level of engagement (concurrent: *while* participating in the discussion vs sequential: *instead of* participating in the discussion). Participants rated perceived intent and the extent to which they would engage in conflict and turnover because of the situation and provided ratings of their own dark core and demographics. |
|  |  | (*b*) *Cohort study*—For matched studies, give matching criteria and number of exposed and unexposed  *Case-control study*—For matched studies, give matching criteria and the number of controls per case | N/A |  |
| Variables | 7 | Clearly define all outcomes, exposures, predictors, potential confounders, and effect modifiers. Give diagnostic criteria, if applicable | Measures (Lines 201 – 219) | **Measures**  **Dark Core.**  The dark core was assessed with the Dirty Dozen (Jonason & Webster, 2010; *α* = 0.95) with 12 items on a scale of 1 (*strongly disagree*) to 5 (*strongly agree*).  **Interpersonal Conflict.**  Conflict was evaluated with 4 items (Spector & Jex, 1998; *α* = 0.89) in which participants were asked to indicate their behavioral frequency on a scale of 1 (*never*) to 5 (*very often*). It includes questions such as “How often do you get into arguments with others at work?”.  **Turnover Intentions***.*  Turnover intentions were assessed with 3 items (Hanish & Hulin, 1991; *α* = 0.85) asking participants to indicate the frequency that they engaged in each behavior on a scale of 1 (*never*) to 5 (*always*) if they were at the job where they observed the coworker described. It includes items such as “It is very likely that I will leave my job”.  **Intent.**  This was measured with a single item created and used by Sliter et al (2015) which asked participants “Was harm intended by the coworker”. Responses included 1 (no intent to harm), 2 (ambiguous intent to harm), and 3 (clear intent to harm).  **Demographics***.*  Age, gender, ethnicity, and employment status was assessed for all participants. |
| Data sources/ measurement | 8* | For each variable of interest, give sources of data and details of methods of assessment (measurement). Describe comparability of assessment methods if there is more than one group | Measures (Lines 201 – 216) | **Measures**  **Dark Core.**  The dark core was assessed with the Dirty Dozen (Jonason & Webster, 2010; *α* = 0.95) with 12 items on a scale of 1 (*strongly disagree*) to 5 (*strongly agree*).  **Interpersonal Conflict.**  Conflict was evaluated with 4 items (Spector & Jex, 1998; *α* = 0.89) in which participants were asked to indicate their behavioral frequency on a scale of 1 (*never*) to 5 (*very often*). It includes questions such as “How often do you get into arguments with others at work?”.  **Turnover Intentions***.*  Turnover intentions were assessed with 3 items (Hanish & Hulin, 1991; *α* = 0.85) asking participants to indicate the frequency that they engaged in each behavior on a scale of 1 (*never*) to 5 (*always*) if they were at the job where they observed the coworker described. It includes items such as “It is very likely that I will leave my job”.  **Intent.**  This was measured with a single item created and used by Sliter et al (2015) which asked participants “Was harm intended by the coworker”. Responses included 1 (no intent to harm), 2 (ambiguous intent to harm), and 3 (clear intent to harm).  **Demographics***.*  Age, gender, ethnicity, and employment status was assessed for all participants. |
| Bias | 9 | Describe any efforts to address potential sources of bias | Lines 188 – 189 | … after accounting for data quality issues using Mahalanobis distance and longstring analysis (*n*= 101; Meade & Craig, 2012). |
| Study size | 10 | Explain how the study size was arrived at | Lines 184 - 186 | An a priori G*Power indicated a sample size of 486 would be sufficient for this study (linear multiple regression with *f^2^*= .03, α= .05, 1-β= .80, with 7 tested predictors, and 8 total number of predictors collected). |

Continued on next page

| Quantitative variables | 11 | Explain how quantitative variables were handled in the analyses. If applicable, describe which groupings were chosen and why | 222 | All measured variables scale scores were calculated through an aggregate of the items. |
| --- | --- | --- | --- | --- |
| Statistical methods | 12 | (*a*) Describe all statistical methods, including those used to control for confounding | 223-224  228-229  237-239  247-249 |  |
|  |  | (*b*) Describe any methods used to examine subgroups and interactions | 259-274 |  |
|  |  | (*c*) Explain how missing data were addressed | 223 |  |
|  |  | (*d*) *Cohort study*—If applicable, explain how loss to follow-up was addressed  *Case-control study*—If applicable, explain how matching of cases and controls was addressed  *Cross-sectional study*—If applicable, describe analytical methods taking account of sampling strategy | N/A |  |
|  |  | (*e*) Describe any sensitivity analyses | N/A |  |
| Results | | | | |
| Participants | 13* | (a) Report numbers of individuals at each stage of study—eg numbers potentially eligible, examined for eligibility, confirmed eligible, included in the study, completing follow-up, and analysed | 187 – 192 |  |
|  |  | (b) Give reasons for non-participation at each stage | 188 – 189 |  |
|  |  | (c) Consider use of a flow diagram | N/A |  |
| Descriptive data | 14* | (a) Give characteristics of study participants (eg demographic, clinical, social) and information on exposures and potential confounders | 189 – 192 |  |
|  |  | (b) Indicate number of participants with missing data for each variable of interest | N/A |  |
|  |  | (c) *Cohort study*—Summarise follow-up time (eg, average and total amount) | N/A |  |
| Outcome data | 15* | *Cohort study*—Report numbers of outcome events or summary measures over time |  |  |
|  |  | *Case-control study—*Report numbers in each exposure category, or summary measures of exposure |  |  |
|  |  | *Cross-sectional study—*Report numbers of outcome events or summary measures | *Table 1* |  |
| Main results | 16 | (*a*) Give unadjusted estimates and, if applicable, confounder-adjusted estimates and their precision (eg, 95% confidence interval). Make clear which confounders were adjusted for and why they were included | Table 2 and Table 3 |  |
|  |  | (*b*) Report category boundaries when continuous variables were categorized | N/A |  |
|  |  | (*c*) If relevant, consider translating estimates of relative risk into absolute risk for a meaningful time period | N/A |  |

Continued on next page

| Other analyses | 17 | Report other analyses done—eg analyses of subgroups and interactions, and sensitivity analyses | N/A |  |
| --- | --- | --- | --- | --- |
| Discussion | | | | |
| Key results | 18 | Summarise key results with reference to study objectives | 280 – 322 |  |
| Limitations | 19 | Discuss limitations of the study, taking into account sources of potential bias or imprecision. Discuss both direction and magnitude of any potential bias | 323 – 354 |  |
| Interpretation | 20 | Give a cautious overall interpretation of results considering objectives, limitations, multiplicity of analyses, results from similar studies, and other relevant evidence | 280 – 322 |  |
| Generalisability | 21 | Discuss the generalisability (external validity) of the study results | 280 – 322 |  |
| Other information | |  | | |
| Funding | 22 | Give the source of funding and the role of the funders for the present study and, if applicable, for the original study on which the present article is based | N/A |  |

*Give information separately for cases and controls in case-control studies and, if applicable, for exposed and unexposed groups in cohort and cross-sectional studies.

**Note:** An Explanation and Elaboration article discusses each checklist item and gives methodological background and published examples of transparent reporting. The STROBE checklist is best used in conjunction with this article (freely available on the Web sites of PLoS Medicine at http://www.plosmedicine.org/, Annals of Internal Medicine at http://www.annals.org/, and Epidemiology at http://www.epidem.com/). Information on the STROBE Initiative is available at www.strobe-statement.org.
